# Workplace Violence and Turnover Intention Among Psychiatrists in a National Sample in China: The Mediating Effects of Mental Health

**DOI:** 10.3389/fpsyt.2022.855584

**Published:** 2022-06-15

**Authors:** Yanhua Chen, Peicheng Wang, Lina Zhao, Yanrong He, Nuoya Chen, Huanzhong Liu, Yuanli Liu, Tingfang Liu, Yi-lang Tang, Feng Jiang, Jiming Zhu

**Affiliations:** ^1^Vanke School of Public Health, Tsinghua University, Beijing, China; ^2^School of Medicine, Tsinghua University, Beijing, China; ^3^Institute for Hospital Management, Tsinghua University, Beijing, China; ^4^Health Related Activity Recognition System Based on IoT Project, University of Macerata, Macerata, Italy; ^5^Department of Psychiatry, Chaohu Hospital of Anhui Medical University, Hefei, China; ^6^Anhui Psychiatric Center, Anhui Medical University, Hefei, China; ^7^School of Health Policy and Management, Chinese Academy of Medical Sciences & Peking Union Medical College, Beijing, China; ^8^Mental Health Service Line, Atlanta VA Medical Center, Decatur, GA, United States; ^9^Addiction Psychiatry Fellowship Program, Department of Psychiatry and Behavioral Sciences, Emory University, Atlanta, GA, United States; ^10^School of International and Public Affairs, Shanghai Jiao Tong University, Shanghai, China; ^11^Institute of Healthy Yangtze River Delta, Shanghai Jiao Tong University, Shanghai, China; ^12^Institute for Healthy China, Tsinghua University, Beijing, China

**Keywords:** workplace violence, turnover intention, mental health, psychiatrists, China

## Abstract

**Background:**

Workplace violence (WPV) in healthcare has received much attention worldwide. However, scarce data are available on its impact on turnover intention among psychiatrists, and the possible mechanisms between WPV and turnover intention have not been explored in China.

**Methods:**

A cross-sectional survey was conducted among psychiatrists in 41 tertiary psychiatric hospitals from 29 provinces and autonomous regions in China. A stress-strain-outcome (SSO) model was adopted to examine the effects of WPV on mental health and turnover intention. The association and mediation by burnout and stress were examined by multivariate logistic regression (MLR) and generalized structure equation modeling (GSEM).

**Results:**

We invited 6,986 psychiatrists to participate, and 4,520 completed the survey (64.7% response rate). The prevalence of verbal and physical violence against psychiatrist in China was 78.0 and 30.7%, respectively. MLR analysis showed that psychiatrists who experienced verbal violence (OR = 1.15, 95% CI = 1.10–1.21) and physical violence (OR = 1.15, 95% CI = 1.07–1.24) were more likely to report turnover intention. GSEM analysis showed that burnout (β = 4.00, *p* < 0.001) and stress (β = 1.15, *p* < 0.001) mediated the association between verbal violence and turnover intention; similarly, burnout (β = 4.92, *p* < 0.001) and stress (β = 1.80, *p* < 0.001) also mediated the association between physical violence and turnover intention.

**Conclusions:**

Experience of WPV is a significant contributor to turnover intention among psychiatrists. Mental health status, such as burnout and stress level significantly mediated the association. Policy makers and hospital administrators need to be aware of this association. Action is needed to promote mental health among the psychiatrists to improve morale and workforce sustainability.

## Introduction

Although some progress has been made in recent years, including the improved quality of training programs (1), the psychiatric workforce is still in severe shortage (2) and unevenly distributed in China. Maintaining workforce sustainability and reducing attrition are vital ways to slow down the shortage of mental health workforce (3). To reduce turnover rate for psychiatrists, it is important to identify the upstream drivers of turnover intention of psychiatrists, and then to inform strategies in recruitment and retention.

In recent decades, workplace violence (WPV) against healthcare workers has drawn attention and become a serious public health issue globally (4, 5). Workplace violence can be defined as “incidents where staff are abused, threatened or assaulted in circumstances related to their work, involving an explicit or implicit challenge to their safety, wellbeing or health” (6). According to the World Health Organization (WHO), health workers are subject to high risk of violence, with 8%-38% of health workers suffering from physical violence at some point in their careers, and even more are threatened by or exposed to verbal aggression (7). It has been found that healthcare workers in the psychiatric settings are approximately three times higher of encountering violence (8, 9) when compared to other medical specialties. Patients' violent behavior, especially physical aggression and verbal violence in psychiatric settings, was of great concern for researchers (10–12) and has become increasingly common in China (13, 14).

WPV can physically and psychologically harm healthcare workers and affect the workforce stability (15). A growing body of research literature suggests that WPV is a key risk factor associated with turnover intention. For instance, a study from an emergency department in Korea showed that WPV increased nurses' intention to leave the hospital (16). A study among physicians in China found that WPV was significantly associated with turnover intention (17). Another survey with physicians in Finland also showed that WPV led to increased turnover intention (18).

The stressor–strain–outcome (SSO) model specified a process between stressors and the outcome with a mediating role played by strain (19, 20). In the SSO model, stressors are environmental stimulus that individuals experience, strains are individuals' personal emotions, and outcomes are behavioral responses to stressors (21). According to the SSO model, strain plays an essential role in the associations between workplace violence and turnover intention. Other related research reaches the consensus that stress and exhaustion are viewed as transactional processes between individual environment stimulus and response behaviors (22, 23). A meta-analytic review proposed that, the negative effects of WPV on affects and attitudes would have indirect consequences on behavioral outcomes (24). Many researchers further examined mental health as a mediator in the association of WPV with turnover intention. Recently, burnout and stress were considered as key mediators and have been examined (25–27). Nonetheless, few of the existing studies systematically explored the relationship between WPV and turnover intention among psychiatrists, and none have examined the mechanism embedded in the stressor-to-outcome process.

With a widely recognized shortage of mental health care workers (28), preventing brain drain has become one of workable solutions to psychiatrists shortage both in short-and long-term. Understanding the impacts of WPV on turnover intention is crucial for preventing more psychiatrists from quitting their job. This is of even more significance in China as a high rate of WPV in healthcare setting has been reported (14). Our study, based on the SSO model, would reveal the transactional process among WPV, mental health and turnover intention in order to provide insight for prevention interventions (22). Based on the SSO model, our research examined the transactional process from WPV to turnover intention by focusing on the following: (1) identifying upstream drivers of turnover intention among psychiatrists; (2) examining the association between verbal and physical violence and turnover intention among psychiatrists; (3) exploring the mediating roles of burnout and stress in the process. The study aimed to understand the mechanism behind turnover intention and shed light on policymaking, in order to maintain human resource sustainability in psychiatric settings.

## Methods

### Participants and Procedures

Data were retrieved from 2019 National Hospital Performance Evaluation Survey, which approached 41 tertiary psychiatric hospitals in 29 provinces across China. Only two provinces, namely Gansu and Tibet, were not covered by the survey as there were no tertiary psychiatric hospitals there. All the psychiatrists (*n* = 6,986) from the 41 hospitals were invited to participate in this survey and 4,520 (64.7%) completed the questionnaire. The survey was conducted anonymously and voluntarily through WeChat, an online social media application widely used in China. An electronic informed consent form was obtained from each participant before they began answering questions. Each WeChat account was allowed to submit the questionnaire once only. Socio-demographic data, self-reported experience of workplace violence, turnover intention and mental health status were collected. The research protocol was approved by the Ethics Committee of Chaohu Hospital of Anhui Medical University (No. 201903-kyxm-02).

### Measures

#### Burnout

Burnout was measured with the Chinese version of the Maslach Burnout Inventory-Human Service Survey (MBI-HSS). The MBI-HSS has been used in many studies including in Chinese samples (29). It has 22 items and measures burnout from three dimensions: emotional exhaustion (EE, 9 items), depersonalization (DP, 5 items) and reduced personal accomplishment (PA, 8 items). A seven-point scale ranging from 0 (“never experienced such a feeling”) to 6 (“experience such feelings every day”) was used to measure how much respondents agree or disagree with a particular statement. In this study, higher scores (ranging from 0 to 84) in emotional exhaustion and depersonalization subscales indicate higher degree of burnout (30, 31), and the Cronbach alpha coefficient for reliability testing was 0.926.

#### Stress

Stress was measured by the Depression Anxiety Stress Scale (DASS, 21 items). DASS-21 was a quantitative measure that examines three domains: depression, anxiety, and stress. Seven items scored from 0 (did not apply to me at all) to 3 (applied to me very much or most of the time) were calculated within each domain. The scale has been used as a screening tool for different populations with various culture background (32) as well as for Chinese samples (33). Based on the scale, the stress score in this study ranged from 0 to 21. The DASS-21 serves as a short version of the DASS (42 items). The final score of each scale was multiplied by two (ranging from 0 to 42), making the scores comparable with the normal DASS scores (34, 35). The Cronbach alpha reliability coefficients (α = 0.956; depression α =0.912; anxiety α = 0.877; and stress α =0.889) for DASS subscales indicates the reliability of our sample for further statistical analysis.

#### Workplace Violence

Participants were asked to answer two questions concerning the verbal and physical violence they experienced in the workplace. The first question is “How many times, in the past 12 months, did you find yourself in a situation of verbal aggression (e.g., expressions of abuse, slandering, contempt, insulting or humiliating without physical contact) by patients?” The second question is “How many times, in the past 12 months, did you find yourself in a situation of physical aggression by patients (e.g., pushing, hitting, inflicting physical harm on persons or violence with weapons)?” Answer score: 1 = never/almost never, 2 = <12 times/year, 3 = once a month, 4 = 2–3 times/month, 5 = once a week, 6 = 2–5 times/week, 7 = almost every day. Higher scores indicates a higher level of experienced violence (3, 36).

#### Turnover Intention

Psychiatrists' turnover intention was evaluated by a single-item question asking “Have you ever thought of quitting your job in the past month?” and the response included “yes” and “no.”

#### Demographic Factors

In this study we included socio-demographic and occupational characteristics data such as, age, gender (male or female), education level (associate degree or lower, college degree, or master's degree or higher), working years (<5, 5–9,10–19 or ≥20 years old), monthly net income (<5,001, 5,001–8,000, 8,001–12,000, or more than 12,000, in CNY), working hours per week (<41, 41–48, 49–54, ≥55), and self-rated health status (unsatisfied, somewhat satisfied, or satisfied).

### Data Analysis

The multivariable logistic regression (MLR) model was used to examine the role played by demographic factors, stress, burnout and WPV in causing widespread turnover intention. To assess the mediating roles played by burnout and stress on the relationship between WPV and turnover intention, generalized structural equation models (GSEM) were constructed for verbal and physical violence by specifying a binomial distribution of outcome variabls, and then a logit link was used to estimate the odds ratios (ORs) and corresponding 95% confidence intervals (CI). Harman's single factor test was performed to address common method bias issue. Mediation analysis was supplementarily used to test indirect effects in our model. Root mean squared error of approximation (RMSEA) and standardized root mean squared residual (SRMR) were assessed for the GSEM model's fit. Given that the DASS-21 is a widely used comprehensive assessment scale for mental health disorders, DASS-Depression and DASS-Anxiety were used for robustness check to verify our findings. All analyses were done with STATA (version 16) and Mplus 7.0. Two-tailed tests with *P*-value < 0.05 were considered statistically significant.

## Results

### Participants' Characteristics

The sociodemographic characteristics and the basic profiles of 4,520 psychiatrists are shown in Table 1. The mean age of the participants was 38.5 (SD = 8.6); 41.9% were male, and 97.3% had a college degree (including a medical degree) or above. The median income was 7,000 RMBs (~ $1,082), with only 36.7% of them earned more than 8,000 RMBs (~$1,236). The mean working hours per week was 53.0 h (SD = 16.2), and 37.0% of the participants worked more than 55 h per week. Overall, the rate of turnover intention was 31.9% in this sample.

**Table 1:** Characteristics of the participants (*n* = 4,520).

**Characteristics**	**Psychiatrists**
Age, mean (SD), years	38.5 (8.6)
<30	602 (13.3)
30–39	2,207 (48.8)
40–49	1,082 (23.9)
≥50	629 (13.9)
Gender
Male	1,892 (41.9)
Female	2,628 (58.1)
Education level
Associate degree or less	123 (2.7)
College degree	2,866 (63.4)
Master's degree or above	1,531 (33.9)
Working years, years
<5	754 (16.7)
5–9	954 (21.1)
10–19	1,548 (34.2)
≥20	1,264 (28.0)
Income, median (IQR), RMBs^a^	7,000 (5,000–10,000)
<5,001 (low)	1,325 (29.3)
5,001–8,000 (middle)	1,536 (34.0)
8,001–12,000 (high)	1,010 (22.3)
≥12,001 (very high)	649 (14.4)
Working hours per week, mean (SD), hours	53.0 (16.2)
<41	1,229 (27.2)
41–48	914 (20.2)
49–54	706 (15.6)
≥55	1,671 (37.0)
Self-rated health
Unsatisfied	2,164 (47.9)
Fair	1,813 (40.1)
Satisfied	543 (12.0)
Burnout, mean (SD)	26.5 (17.4)
Stress, mean (SD)	10.9 (7.9)

a *In 2019, US $ 1 was equivalent to 6.47 RMBs*.

### Prevalence of Workplace Violence

As Table 2 shows, when compared with physical violence (30.7%), a greater proportion of psychiatrists in China experienced verbal violence (78.0%). Specifically, verbal violence was more common in males (80.1%), among people in their 40s (81.3%), with a master's degree or above (80.5%), in the highest income category (82.4%), or among those who worked 49–54 h per week (84.3%). Physical violence was most common in males (35.5%), among those in their 30s (32.4%), with a college degree (32.2%), in the middle-income category (33.3%), or among those who worked 49–54 h per week (36.1%).

**Table 2:** The prevalence of exposure to workplace violence among psychiatrists.

**Variables**	**Verbal violence *n* (%)**	**Physical violence *n* (%)**
Total	3,524 (78.0)	1,386 (30.7)
Gender
Male	1,515 (80.1)	672 (35.5)
Female	2,009 (76.4)	714 (27.2)
χ^2^	8.43**	36.07***
Age
<30	428 (71.1)	156 (25.9)
30–39	1,774 (80.4)	715 (32.4)
40–49	880 (81.3)	349 (32.3)
≥50	442 (70.3)	166 (26.4)
χ^2^	52.84***	16.20**
Education level
Associate degree or less	67 (54.5)	19 (15.4)
College degree	2,225 (77.6)	924 (32.2)
Master's degree or above	1,232 (80.5)	443 (28.9)
χ^2^	45.29***	18.90***
Income level
<5,001 (low)	972 (73.4)	394 (29.7)
5,001–8,000 (middle)	1,197 (77.9)	512 (33.3)
8,001–12,000 (high)	820 (81.2)	315 (31.2)
≥12,001 (very high)	535 (82.4)	165 (25.4)
χ^2^	30.02***	14.20**
Working hours per week
<41	801 (65.2)	275 (22.4)
41–48	722 (79.0)	264 (28.9)
49–54	595 (84.3)	255 (36.1)
≥55	1,406 (84.1)	592 (35.4)
χ^2^	171.07***	68.79***

**p < 0.01; ***p < 0.001. The proportion was calculated as the number of people of certain characteristics divided by the total number of psychiatrists within each socio-demographic group. Workplace violence was a dummy variable that participants have experienced violence at least once in the past 12 months.

### Factors Associated With Turnover Intention

Table 3 presents the results of the MLR model. Male, working for 10-19 years, working more than 48 h per week were factors that are more likely to be related with turnover intention. Participants with higher monthly income and more satisfied self-reported health status were less likely to have turnover intention. Model 2–5 suggested the association between verbal violence, physical violence, burnout, and stress with turnover intention after controlling for all the covariates. Exposure to verbal violence (OR = 1.15, 95% CI = 1.10–1.21) and physical violence (OR = 1.15, 95% CI = 1.07–1.24) were significantly associated with turnover intention. In addition, burnout (OR = 1.05, 95% CI = 1.04–1.05) and stress (OR = 1.07, 95% CI = 1.076–1.08) would significantly increase the odds of psychiatrists' turnover intention. As the direct effect of WPV was insignificant, we further conducted the moderation analysis (see Supplementary Table 1). The results showed that WPV had no significant moderating effect on the association between mental health and turnover intention. We also examined the association between two subscales of DASS (depression and anxiety) and turnover intention as robustness test (see Supplementary Table 2).

**Table 3:** Logistics regression analyses for factors associated with turnover intention.

	**Model 1**	**Model 2**	**Model 3**	**Model 4**	**Model 5**
Verbal violence		**1.15 (1.10–1.21)**			
Physical violence			**1.15 (1.07–1.24)**		
Burnout				**1.05 (1.04–1.05)**	
Stress					**1.07 (1.06–1.08)**
Age	0.98 (0.96, 1.00)	0.98 (0.96–1.00)	0.98 (0.96–1.00)	0.99 (0.97–1.01)	0.98 (0.96–1.00)
Gender
Male	Ref.	Ref.	Ref.	Ref.	Ref.
Female	**0.87 (0.76, 1.00)**	0.88 (0.77–1.00)	0.90 (0.78–1.03)	0.90 (0.76–1.03)	0.90 (0.78–1.03)
Education
Associate degree or less	Ref.	Ref.	Ref.	Ref.	Ref.
College degree	1.59 (0.97, 2.58)	1.54 (0.94–2.51)	1.54 (0.94–2.50)	**1.72 (1.01–2.93)**	1.56 (1.94–2.59)
Master's degree or above	1.55 (0.94, 2.57)	1.51 (0.91–2.51)	1.52 (0.92–2.52)	**1.70 (0.98–2.94)**	1.61 (0.95–2.72)
Working years
<5	Ref.	Ref.	Ref.	Ref.	Ref.
5–9	1.10 (0.87, 1.39)	1.06 (0.84–1.34)	1.08 (0.86–1.37)	1.04 (0.81–1.33)	1.08 (0.85–1.38)
10–19	**1.58 (1.21, 2.08)**	**1.51 (1.15–1.99)**	**1.56 (1.19–2.05)**	**1.39 (1.04–1.86)**	**1.59 (1.20–2.10)**
≥20	1.56 (0.99, 2.47)	1.50 (0.94–2.38)	1.53 (0.97–2.43)	1.41 (0.86–2.30)	**1.60 (1.00–2.58)**
Income
<5,001	Ref.	Ref.	Ref.	Ref.	Ref.
5,001–8,000	0.96 (0.81, 1.13)	0.95 (0.80–1.12)	0.96 (0.81–1.14)	0.96 (0.81–1.15)	1.02 (0.86–1.21)
8,001–12,000	0.84 (0.70, 1.03)	0.84 (0.69–1.02)	0.85 (0.70–1.03)	0.86 (0.70–1.06)	0.95 (0.78–1.16)
≥12,001	**0.60 (0.47, 0.77)**	**0.60 (0.47–0.77)**	**0.61 (0.48–0.78)**	**0.61 (0.47–0.79)**	**0.70 (0.55–0.90)**
Working hours per week
<41	Ref.	Ref.	Ref.	Ref.	Ref.
41–48	1.19 (0.97, 1.45)	1.13 (0.92–1.39)	1.17 (0.95–1.43)	1.04 (0.84–1.29)	1.11 (0.90–1.37)
49–54	**1.36 (1.09, 1.69)**	**1.27 (1.02–1.59)**	**1.32 (1.06–1.64)**	1.10 (0.87–1.38)	**1.28 (1.03–1.60)**
≥55	**1.78 (1.50, 2.13)**	**1.66 (1.39–1.98)**	**1.72 (1.44–2.05)**	**1.33 (1.10–1.60)**	**1.52 (1.27–1.83)**
Self-rated health
Unsatisfied	Ref.	Ref.	Ref.	Ref.	Ref.
Fair	**0.47 (0.41, 0.54)**	**0.49 (0.42–0.56)**	**0.48 (0.42–0.55)**	**0.70 (0.60–0.81)**	**0.62 (0.54–0.72)**
Satisfied	**0.20 (0.15, 0.26)**	**0.22 (0.16–0.29)**	**0.21 (0.16–0.27)**	**0.41 (0.31–0.55)**	**0.34 (0.25–0.45)**

*Boldface indicates statistical significance (p < 0.05). Ref., reference*.

### Mediating Effects of Burnout and Stress

**Figure 1:**
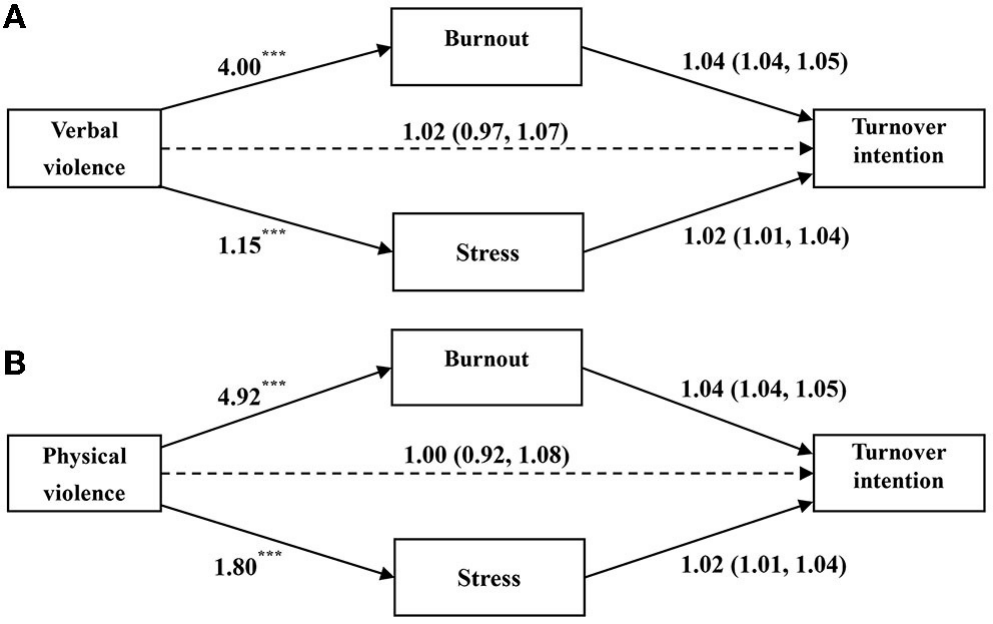
Mediation effects of mental health between WPV and turnover intention. **(A)** illustrates the path coefficients of the relationship between verbal violence and turnover intention; **(B)** of the relationship between physical violence and turnover intention. Regression coefficients are shown for the path from WPV to burnout and stress, *p < 0.05; **p < 0.01; ***p < 0.001. ORs and corresponding 95% CI (in parentheses) are shown for the path between mental health and turnover intention, which is measured as a binary variable. Both models adjusted for age, gender, education, working years, income, working hours per week, self-rated health.

In Figure 1A, the GSEM results demonstrated that verbal violence predicted burnout (β = 4.00, *P* < 0.001) and stress (β = 1.15, *P* < 0.001) directly and positively, but verbal violence had no significantly direct effects on turnover intention (OR = 1.02, 95% CI = 0.97, 1.07). Figure 1B showed that physical violence also had a direct and significant predictive effect on burnout (β = 4.92, *P* < 0.001) and stress (β = 1.80, *P* < 0.001); meanwhile, physical violence had no significantly indirect effects on turnover intention (OR = 1.00, 95% CI = 0.92, 1.08). In both models, burnout (OR = 1.04, 95% CI = 1.04, 1.05) and stress (OR = 1.02, 95% CI = 1.01, 1.04) were significant predictors of turnover intention. The results of robustness check for depression and anxiety were showed in Supplementary Figures 1, 2.

The mediation analysis was used to test the significance of mediating effects of burnout and stress. The 95% confidence intervals for burnout and stress did not contain 0, indicating that the mediating effects were significant. The results in Table 4 suggest that the mediating effects of verbal violence (OR = 0.20, 95% CI = 0.17–0.22) and physical violence (OR = 0.25, 95% CI = 0.22–0.29) over turnover intention were statistically significant. The mediating effect of “Verbal violence → Burnout → Turnover intention” was 0.17 (95% CI = 0.14–0. 19); and the effect of “Physical violence → Stress → Turnover intention” was 0.03 (95% CI = 0.01–0.04). The mediating effect of “Physical violence → Burnout → Turnover intention” was 0.21 (95% CI = 0.17–0.24); and the effect of “Physical violence → Stress → Turnover intention” was 0.06 (95% CI = 0.02–0.06). Indirect effects of depression and anxiety were tested (see Supplementary Table 3). In addition, under the linear assumption, RMSEA and SRMR were assessed with a large sample size used in this study (37–39). The test model fit both conducted in GSEM model A (RMSEA = 0.066 and SRMR = 0.082) and GSEM model B (RMSEA = 0.064 and SRMR = 0.081), indicating an adequate model fit for our GSEM models.

**Table 4:** Mediation analysis of indirect effects of burnout and stress.

**Indirect effects**	**Effects size**	**95% CI**
Verbal violence → Turnover intention	0.20	(0.17, 0.22)
Verbal violence → Burnout → Turnover intention	0.17	(0.14, 0.19)
Verbal violence → Stress → Turnover intention	0.03	(0.01, 0.04)
Physical violence → Turnover intention	0.25	(0.22, 0.29)
Physical violence → Burnout → Turnover intention	0.21	(0.17, 0.24)
Physical violence → Stress → Turnover intention	0.04	(0.02, 0.06)

*Both models adjusted for age, gender, education, working years, income, working hours per week, self-rated health*.

## Discussion

With a large national sample of psychiatrists from 41 tertiary psychiatric hospitals in China, we found that psychiatrists experienced higher rates of verbal violence (78.0%) and physical violence (30.7%) when compared to the overall rates of verbal violence (48.52%) and physical violence (5.84%) in 144 Chinese public hospitals (40). Some possible reasons may explain the high rates. In psychiatric settings, patients with unstable mental status may be stressed and are more likely to express anger toward those who care for them. In addition, due to the increasing demand of mental health services in China, psychiatrists are facing an inflow of patients suffering from mental illness, which would increase their workload. Hospitalizations in psychiatric settings maybe for days, weeks, or longer, and this ongoing care relationship may also increase the incidence of WPV (41–43). The prevalence of WPV vary across socio-economic groups. This finding is consistent with our other study (44), where we found that WPV undermines workforce stability and got inspired to further explore the mechanisms between WPV and turnover intention. In this study, we examined the associations between workplace violence and turnover intention, and the mediating effects of mental health on the associations, contributing to the existing literature on WPV, mental health and turnover intention among physicians. Our findings suggest the existence of a psychologically transactional process (as strains) between WPV (as individual environment stimulus factors) and turnover intention (as response behaviors) among psychiatrists.

We found a high rate (31.9%) of psychiatrists reporting they would leave their job. The percentage was also much higher than the turnover intention of nurses (20.0%) and pharmacists (17.8%) in psychiatric settings (1, 45). Consistent with previous studies (46–48) in several countries, psychiatrists with higher salary and satisfaction with their health conditions are more likely to stay in their current job; male doctors with longer work history and working hours are more likely to have turnover intention. These findings may help policymakers and hospital administrator to take more targeted measures (e.g., health promotion, lower hours of work or higher level of salary) to retain employees and boost their wellbeing. In addition, consistent with previous studies, healthcare workers who reported burnout and stress were more likely to have turnover intentions (49–51). This finding suggests that reducing burnout and stress among psychiatrists may be effective in preventing their turnover intention.

We found that psychiatrists with greater exposure to WPV were more likely to have turnover intention. This is consistent with previous studies in which workplace violence was found to be associated with higher turnover intention among physicians (52, 53). As the experience of verbal violence and physical violence were positively associated with leave intention, it was not surprising that such “negative experience” could contribute to the increasing turnover intention. Concordant with previous studies where physical and verbal violence are found to have significant impact on burnout among Chinese physicians (40), our findings suggest that WPV, including both verbal violence and physical violence, is associated with burnout and stress. A study in Israel also suggests that verbal and physical violence were significantly associated with stress among mental health nurses (10).

Moreover, the mediation analysis in this study showed that the relationship between WPV and turnover intention was mediated by mental health. This result supports the mediating effect of mental health (including occupational stress and burnout) reported in association between patient violence and turnover intention among nurses (25). Our findings were also partly in line with the observation during COVID-19 in China that the effect of workplace violence on turnover intention was partially mediated by mental health (i.e., stress) (27). Although exposure to WPV might lead to mental health problems, our analysis indicates that mental health is a meaningful knob to reduce the negative impact of WPV on turnover intention in this population.

Strategies are needed to reduce and prevent the incidence of WPV (54, 55). Adequate training, education and support should be provided for healthcare workers (56). Improving communication skills of healthcare workers would also be helpful in reducing patient-physician misunderstandings (57). Legislation against WPV in healthcare facilities can be established in order to protect vulnerable healthcare workers (9). Governments could declare a crackdown on assault on doctors and on violence in hospitals (14). Our findings additionally suggest that hospital administrators should take measures to promote the mental health and wellbeing of psychiatrists after the exposure to WPV. This indicates that, besides providing sufficient support, helping psychiatrists deal with mental health in the face of WPV might be equally important to reduce their turnover intention (58). Just as researchers advocated, hospital management should consider the effects of workplace violence on workers' mental health and their subsequent intention to leave (59). The de-escalation of incidents would be an effective way to decrease the likelihood of a violent outcome (60). For example, the hospital management policy should conduct stress reduction intervention program to mitigate mental health burden (10). Hospitals can also provide specialized and confidential trauma-informed mental health services (61) and provide timely psychological interventions (such as establishing professional pressure relief workshops), and arrange psychologists for physicians who have experienced workplace violence (58).

The study has a few limitations. First, the data was collected in a self-reported manner, which might have cognitive bias. Second, the data sets were cross-sectional with no causal relationship can be inferred. Third, psychological violence and sexual harassment and other implicit violence were not included in our survey. While we discussed the mediation effects of burnout and stress, more potential mediators (e.g., job performance, professional identity, and job security) between WPV and turnover intention could be further explored. Fourth, as STATA does not provide GSEM model fit information, we assessed RMSEA and SRMR with a large sample size under the linear assumption instead. Finally, the study focused primarily on violence from patients and their families and did not evaluate violence from staff members, which could have similar, if not more, effects on individuals who were exposed to it.

## Conclusion

It is alarming that nearly one third of psychiatrists in China have reported an intention to leave their current position. We have also found that WPV, including verbal and physical violence, are significant predictors of turnover intention, while mental health has significant mediating effects on turnover intention. Our findings suggest, while measures to decrease WPV are important, policy makers and hospital administrators need to be aware of the mediating role of mental health on psychiatrists. It may be helpful and cost-effective to promote mental health among the psychiatrists to improve their morale and workforce sustainability.

## Data Availability Statement

Data are available from the corresponding authors upon reasonable request.

## Ethics Statement

The studies involving human participants were reviewed and approved by the Ethics Committee of Chaohu Hospital of Anhui Medical University (No. 201903-kyxm-02). The Ethics Committee waived the requirement of written informed consent for participation.

## Author Contributions

FJ, HL, TL, and YL collected the data set. The original idea for the research was developed by YC, PW, and JZ. PW, YC, and LZ conducted the statistical analyses. YC, PW, LZ, and JZ drafted the paper. YH, NC, YT, and JZ were involved with manuscript preparation and revisions. All authors approved the final manuscript.

## Conflict of Interest

The authors declare that the research was conducted in the absence of any commercial or financial relationships that could be construed as a potential conflict of interest.

## Publisher's Note

All claims expressed in this article are solely those of the authors and do not necessarily represent those of their affiliated organizations, or those of the publisher, the editors and the reviewers. Any product that may be evaluated in this article, or claim that may be made by its manufacturer, is not guaranteed or endorsed by the publisher.
