# Supplementary material for: Workplace Violence and Turnover Intention Among Psychiatrists in a National Sample in China: The Mediating Effects of Mental Health
Source: Front Psychiatry. 2022 Jun 15;13:855584. doi: 10.3389/fpsyt.2022.855584 (PMC9240432; doi:10.3389/fpsyt.2022.855584)
Supplement: Supplementary file 1 [file Table_1.DOCX]

***Supplementary Material***

# 1 Supplementary Figures and Tables

# 1.1 Supplementary Tables

**Supplementary Table 1.** The moderating effects of workplace violence

|  | Model 1 | |  | Model 2 | |  | Model 3 | |  | Model 4 | |
| --- | --- | --- | --- | --- | --- | --- | --- | --- | --- | --- | --- |
|  | OR (95% CI) | p |  | OR (95% CI) | p |  | OR (95% CI) | p |  | OR (95% CI) | p |
| Verbal violence | 1.076  (0.971, 1.193) | 0.161 |  | **1.188**  **(1.090, 1.295)** | **<0.001** |  | -- | -- |  | -- | -- |
| Physical violence | -- | -- |  | -- | -- |  | 1.071  (0.897, 1.278) | 0.450 |  | 1.132  (0.975, 1.314) | 0.103 |
| Burnout | **1.054**  **(1.045, 1.064)** | **<0.001** |  | -- | -- |  | **1.053**  **(1.044, 1.061)** | **<0.001** |  | -- | -- |
| Stress | -- | -- |  | **1.088**  **(1.068, 1.109)** | **<0.001** |  | -- | -- |  | **1.079**  **(1.061, 1.098)** | **<0.001** |
| Verbal violence 🞨 Burnout | 0.998  (0.995, 1.001) | 0.198 |  | -- | -- |  | -- | -- |  | -- | -- |
| Verbal violence🞨 Stress | -- | -- |  | 0.995  (0.989, 1.000) | 0.056 |  | -- | -- |  | -- | -- |
| Physical violence🞨 Burnout | -- | -- |  | -- | -- |  | 0.998  (0.994, 1.002) | 0.357 |  | -- | -- |
| Physical violence🞨 Stress | -- | -- |  | -- | -- |  | -- | -- |  | 0.997  (0.988, 1.006) | 0.454 |

*Note. Boldface indicates statistical significance (p<0.05). Ref., reference. All models adjusted for age, gender, education, working years, income, working hours per week, self-rated health.*

**Supplementary Table 2.** Logistics regression analyses for factors associated with turnover intention

|  | **Model S1** | **Model S2** |
| --- | --- | --- |
| **Depression** | **1.10 (1.09-1.11)** |  |
| **Anxiety** |  | **1.07 (1.06-1.08)** |
| **Age** | 0.98 (0.96-1.00) | 0.98 (0.96-1.00) |
| **Gender** |  |  |
| Male | Ref. | Ref. |
| Female | 0.95 (0.82-1.10) | 0.90 (0.78-1.03) |
| **Education** |  |  |
| Associate degree or less | Ref. | Ref. |
| College degree | 1.62 (0.96-2.74) | **1.66 (1.00-2.76)** |
| Master’s degree or above | **1.72 (1.00-2.95)** | **1.73 (1.02-2.92)** |
| **Working years** |  |  |
| <5 | Ref. | Ref. |
| 5-9 | 1.06 (0.83-1.35) | 1.06 (0.84-1.35) |
| 10-20 | **1.47 (1.11-1.95)** | **1.52 (1.15-2.00)** |
| ≥20 | 1.48 (0.92-2.39) | 1.47 (0.92-2.34) |
| **Income** |  |  |
| <5001 | Ref. | Ref. |
| 5001-8000 | 1.06 (0.89-1.26) | 1.05 (0.89-1.25) |
| 8001-12000 | 0.98 (0.80-1.20) | 0.98 (0.80-1.19) |
| ≥12001 | **0.74 (0.57-0.95)** | **0.72 (0.56-0.92)** |
| **Working hours per week** |  |  |
| <41 | Ref. | Ref. |
| 41-48 | 1.12 (0.91-1.39) | 1.15 (0.93-1.42) |
| 49-54 | **1.30 (1.04-1.63)** | **1.34 (1.07-1.67)** |
| ≥55 | **1.58 (1.31-1.90)** | **1.59 (1.32-1.90)** |
| **Self-rated health** |  |  |
| Unsatisfied | Ref. | Ref. |
| Fair | **0.66 (0.57-0.77)** | **0.60 (0.52-0.69)** |
| Satisfied | **0.40 (0.30-0.54)** | **0.31 (0.23-0.41)** |

*Note. Boldface indicates statistical significance (p<0.05). Ref., reference.*

**Supplementary Table 3.** Mediation analysis of indirect effects of depression and anxiety

| **Indirect effects** | **Effects size** | **95% CI** |
| --- | --- | --- |
| Verbal violence → Depression → Turnover intention | 0.05 | (0.04, 0.07) |
| Verbal violence → Anxiety → Turnover intention | 0.02 | (0.01, 0.03) |
| Physical violence → Depression → Turnover intention | 0.10 | (0.07, 0.12) |
| Physical violence → Anxiety → Turnover intention | 0.04 | (0.01, 0.06) |

*Note. Both models adjusted for age, gender, education, working years, income, working hours per week, self-rated health.*

# 1.2 Supplementary Figures

**1.05(1.04, 1.06)**

**1.03(1.03, 1.04)**

**4.00^***^**

**1.03 (0.97, 1.08)**

**1.13^***^**

**Verbal violence**

**Burnout**

**Depression**

**Turnover intention**

**(A)**

**1.05(1.04, 1.06)**

**1.03(1.03, 1.04)**

**4.92^***^**

**0.99 (0.91, 1.07)**

**1.93^***^**

**Physical violence**

**Burnout**

**Depression**

**Turnover intention**

**(B)**

**Supplementary Figure 1.** Mediation effects of burnout and depression between WPV and turnover intention

Note: Figure S1A illustrates the path coefficients of the relationship between verbal violence and turnover intention; figure S1B of the relationship between physical violence and turnover intention. Regression coefficients are shown for the path from WPV to burnout and depression, *p<0.05; **p<0.01; ***p<0.001. ORs and corresponding 95% CIs (in parentheses) are shown for the path between mental health and turnover intention, which is measured as a binary variable. Both models adjusted for age, gender, education, working years, income, working hours per week, self-rated health.

**1.02(1.01, 1.03)**

**1.04 (1.04, 1.05)**

**4.00^***^**

**1.02 (0.97, 1.07)**

**1.02^***^**

**Verbal violence**

**Burnout**

**Anxiety**

**Turnover intention**

**(A)**

**1.02(1.01, 1.03)**

**1.05 (1.04, 1.05)**

**4.92^***^**

**0.99 (0.91, 1.07)**

**1.81^***^**

**Physical violence**

**Burnout**

**Anxiety**

**Turnover intention**

**(B)**

**Supplementary Figure 2.** Mediation effects of burnout and anxiety between WPV and turnover intention

Note: Figure S2A illustrates the path coefficients of the relationship between verbal violence and turnover intention; figure S2B of the relationship between physical violence and turnover intention. Regression coefficients are shown for the path from WPV to burnout and anxiety, *p<0.05; **p<0.01; ***p<0.001. ORs and corresponding 95% CIs (in parentheses) are shown for the path between mental health and turnover intention, which is measured as a binary variable. Both models adjusted for age, gender, education, working years, income, working hours per week, self-rated health.
